# Visualizing localized, radiative defects in GaAs solar cells

**DOI:** 10.1038/s41598-022-19187-4

**Published:** 2022-09-01

**Authors:** Behrang H. Hamadani, Margaret A. Stevens, Brianna Conrad, Matthew P. Lumb, Kenneth J. Schmieder

**Affiliations:** 1 National Institute of Standards and Technology, Gaithersburg, MD 20899 USA; 2 NRC Postdoc Residing at NRL, Washington, DC 20375 USA; 3 Formerly With George Washington University, Washington, DC 20052 USA; 4 U.S. Naval Research Laboratory, Washington, DC 20375 USA

**Keywords:** Solar cells, Optical materials and structures

## Abstract

We have used a calibrated, wide-field hyperspectral imaging instrument to obtain absolute spectrally and spatially resolved photoluminescence images in high growth-rate, rear-junction GaAs solar cells from 300 to 77 K. At the site of some localized defects scattered throughout the active layer, we report a novel, double-peak luminescence emission with maximum peak energies corresponding to both the main band-to-band transition and a band-to-impurity optical transition below the band gap energy. Temperature-dependent imaging reveals that the evolution of the peak intensity and energy agrees well with a model of free-to-bound recombination with a deep impurity center, likely a gallium antisite defect. We also analyzed the temperature dependence of the band-to-band transition within the context of an analytical model of photoluminescence and discuss the agreement between the modeling results and external device parameters such as the open circuit voltage of the solar cells over this broad temperature range.

## Introduction

Hyperspectral (HS) imaging in photoluminescence (PL) or electroluminescence (EL) modes is a convenient and powerful technique for spatially resolving defects or regions of heterogeneity across the active layer of a solar cell^1–4^. In HS imaging, every pixel of the captured image, often called an image cube, contains the full spectral information emitted from the corresponding location across the device, hence eliminating the need for performing any secondary spectroscopy to obtain the luminescence spectra. For materials with a single optical transition throughout the active region, HS imaging provides a similar outcome as the traditional, spectrally-insensitive EL or PL imaging. However, when there are several optical transitions present within a material or a device structure, HS luminescence imaging can help construct energy or intensity maps for each of these transitions provided sufficient spectral and spatial resolution exists^3,4^.

Although EL or PL have traditionally been viewed as qualitative imaging techniques, it has been demonstrated in recent years that absolute EL or PL imaging^5^, through a variety of calibration methods, provide rich and quantitative information regarding the features observed in various materials. Such information include quasi-Fermi level splitting energy and photon flux maps^6–8^, lifetime and defect density maps^9^, or even maps of photon flux from different active layers within a stack of multijunction solar cells^10^.

The ability to obtain local spectral information with high resolution (1 µm or less) in wide field becomes particularly interesting when there are localized radiative transitions with peak emission energies close to each other. In most reported studies, high resolution mapping is achieved by micro-PL techniques under a scanning optical microscope^11^. In such cases, the laser intensity is often very high, pushing carrier transport into the high-injection physics limit. Plus, scanning-based imaging is time-consuming and significantly more involved than wide field imaging. Gallium arsenide (GaAs) is one such material where a variety of radiative defects and dislocations have been identified and studied for decades using photoluminescence and other techniques^12,13^. In particular, there are several native-point defect species, such as vacancies in Ga or As sublattices, self-interstitials and antisite defects^14^ that result in radiative recombination at energies different than the band gap energy, *E*_*g*_. Whether such radiative transitions will occur between individual donor–acceptor defect complexes, or between the conduction (or valance) band and an individual impurity band will depend on dopant types and their concentration, growth conditions and other factors^15,16^. In general, the majority of radiative defects in GaAs have been reported as having energies less than the band gap energy; however, extended defects with energies slightly above the band-to-band (BB) transition have also been reported^11^.

GaAs solar cells have shown remarkable performance, owing to their direct band gap, high carrier mobility and a device structure with high material quality and well-studied material and device properties^17^. Recently, rear-junction (RJ) GaAs solar cells with high growth rates (> 1 µm/min) have attracted attention as a realistic target for reducing device cost, currently a significant roadblock to more widespread adoption^18,19^. Comprehensive macroscale electrical characterization of these high growth-rate devices has shown that growth rate and temperature have an impact on defect trap density, non-radiative recombination and the overall external quantum efficiency (EQE) and power conversion efficiency (PCE)^20^. Although the existence of radiative defects in GaAs substrates and films have been studied with PL for decades, there are currently no reports of such defects being visualized with a wide-field, hyperspectral imaging system at the microscale.

In this work, we studied the microscale luminescence properties of RJ GaAs solar cells grown on a production metal organic chemical vapor deposition (MOCVD) tool at a rate of 60 µm/h. The purpose was to gain more insight into localized radiative defect formations and their temperature-dependent characteristics, material pinholes, the heterogeneity of the BB transition and its temperature dependence, the quasi Fermi level splitting and other effects. This task was accomplished using a calibrated, wide-field hyperspectral imaging system in PL mode. The laser excitation source illuminates the device uniformly over the entire field of view, making it possible to correlate absolute PL measurements with external device parameters such as the open circuit voltage, V_oc_. The temperature dependence of the PL flux and peak position of the sub gap defects point to formation of an impurity acceptor band. The temperature behavior of the BB peak can be explained reasonably well with a recent model of PL unifying the band edge and sub-band gap absorption through a unified density-of-states (DOS)-based model with a disordered energy parameter for the tail states^21^. We also extract the external radiative efficiency (ERE) of these devices and show that at temperatures < 140 K, ERE values saturate at ≈ 2%, indicating a radiative upper bound for the performance of these devices as currently structured. In general, the HS luminescence imaging technique provides unique spatial information about defects with very high resolution, and the details provided herein may enable crystal growers to devise improved growth strategies to suppress the formation of these defects.

## Results and discussion

### Localized radiative defects with different emission energies

**Figure 1:**
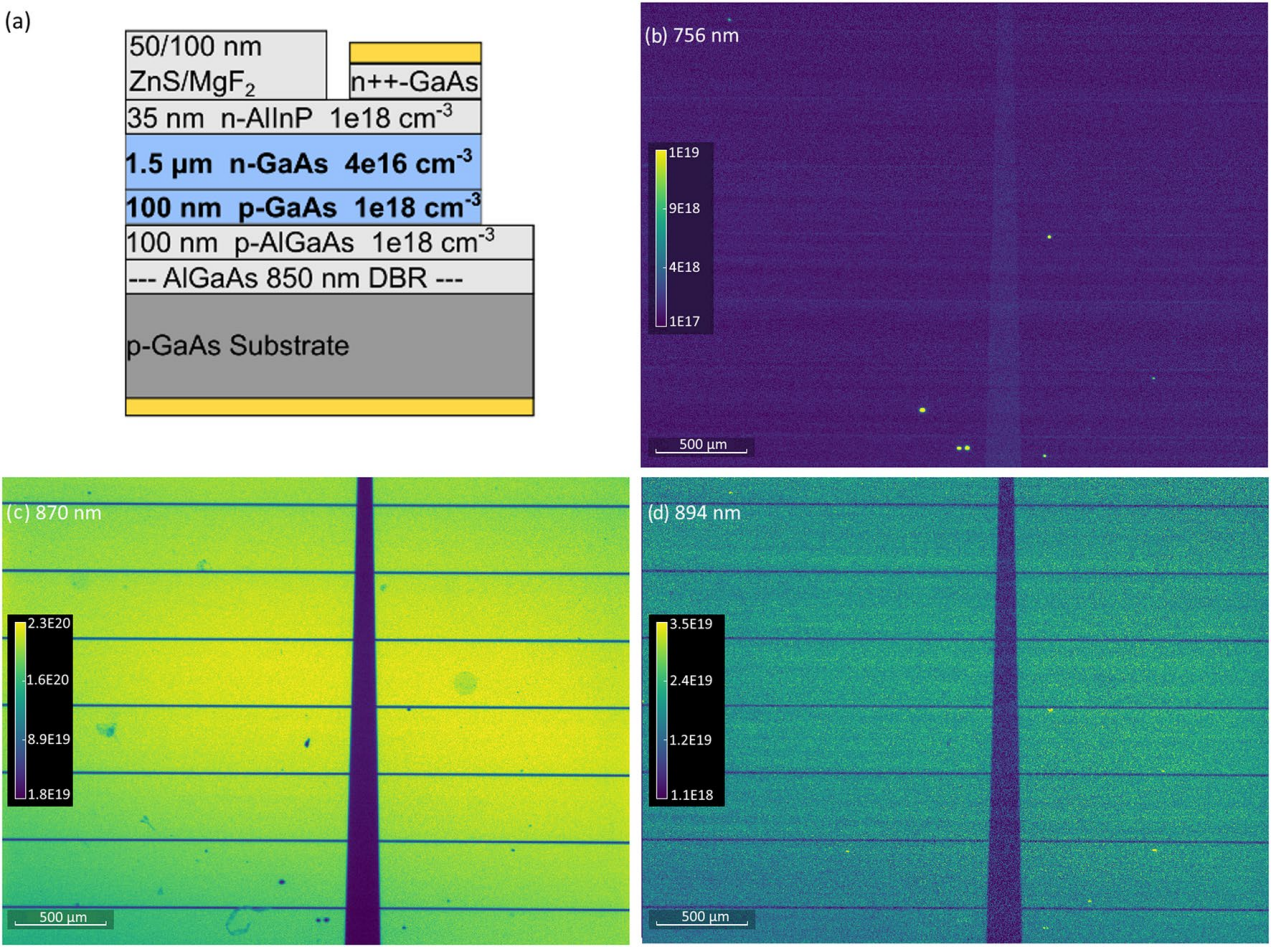
(**a**) Layer structure of the GaAs devices. (**a**–**d**) Absolute PL spectral photon flux maps in photons/(m^2^ s eV) at 300 K and several wavelengths corresponding to the peak emission wavelength of the (**b**) DBR layer, (**c**) BB transition, and (**d**) FB transition.

Figure 1a shows the layer structure of the RJ GaAs devices explored in this work with more details provided in “Methods”. In the RJ architecture, almost all photogenerated carriers are generated in the thick, low-doped n-type GaAs layer. As shown in previous studies^22,23^, this offers a lower dark current than conventional front junction n-on-p devices, and dramatically increases the sensitivity of the device efficiency enhancements due to radiative recombination recycling. The “recycling” effect involves the reabsorption of photons emitted within the device via radiative recombination, resulting in a reduction in the net recombination rate and reduced dark current. An important aspect of this structure is the presence of a high reflectivity back surface to promote reabsorption of photons emitted away from the incident sunlight direction^24^. Our devices were grown on top of an epitaxial distributed Bragg reflector (DBR) to provide high reflectivity near the band edge of GaAs.

Figure 1b–d show a series of absolute PL images (units: photons/m^2^ s eV) under a 5× objective at T = 300 K and wavelengths corresponding to the PL peak maxima of several observed transitions, including (b) 756 nm, (c) 870 nm, and (d) 894 nm. The dark horizontal gridlines and the thicker vertical line are the top electrode contact, with 350 µm spacing between the horizonal lines. The few bright spots appearing in Fig. 1b are consistent with the DBR layer’s PL signal, which we have confirmed in separate, DBR-only films (Supplemental Fig. S1). Therefore, we attribute the emission at 756 nm to emission from the DBR transmitted out of the top layer through pinholes within the film, and not the GaAs active material. The emission at 870 nm (1.425 eV) is attributed to the fundamental band gap E_g_ (300 K) of GaAs (i.e., the BB transition), and although the device contains a doped GaAs layer, the band gap energy is unaffected by Moss–Burstein effects at the low n-doping concentrations in this device^25^. The penetration depth of the 532-nm laser used for these PL measurements is approximately 135 nm, therefore mostly interrogating the low-doped n-GaAs region of these cells. We observe a relatively uniform response across the image except at the location of the pinholes or a few other local defects appearing as darker blue in Fig. 1c. At 894 nm (Fig. 1d), the BB signal is low but a few bright spots appear at locations mostly (but not all) corresponding to the pinhole sites. The signal at these sites is very weak near room temperature but becomes significantly stronger at lower temperatures. The unique PL signal emitted from these sites is consistent with emission from free-to-bound (FB) recombination. Further data supporting the rationale behind attributing this transition as FB will be provided below in the temperature dependence studies of the device.

**Figure 2:**
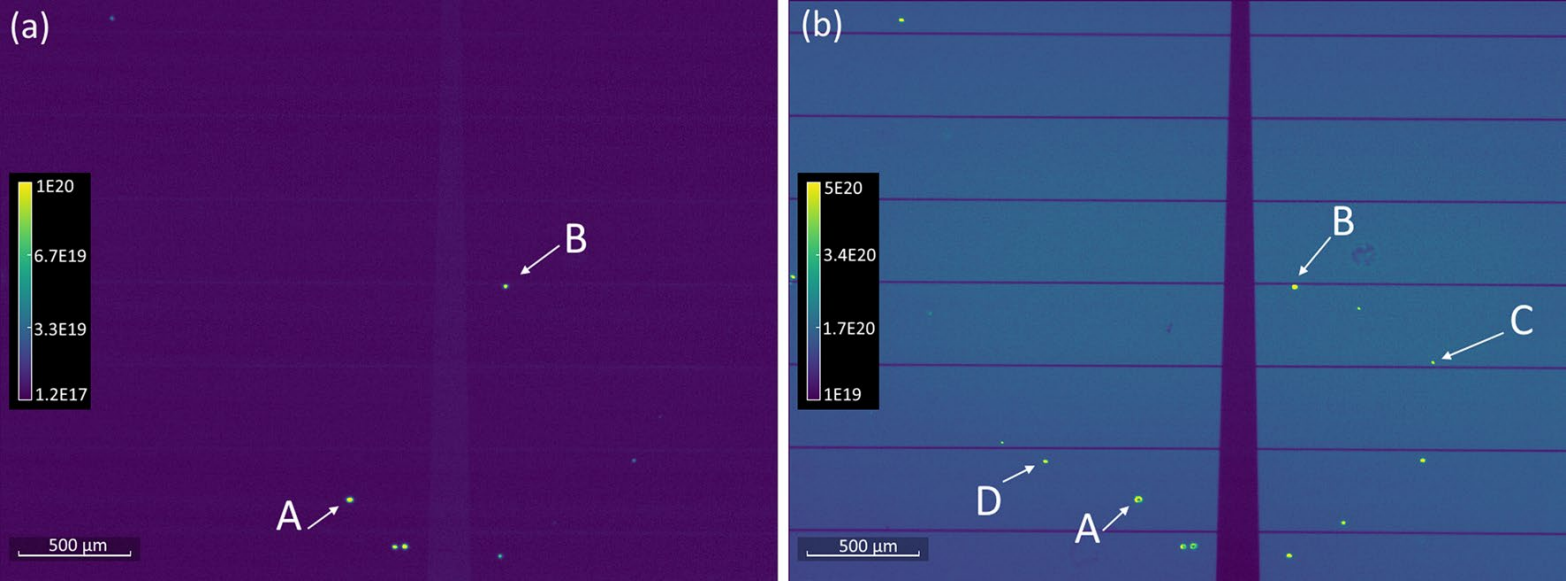
Photon flux maps in photons/(m^2^ s eV) at 77 K and *either* (**a**) 730 nm, or (**b**) 846 nm, corresponding to the DBR and FB peak transitions. The immediate area around pinhole defects visible in (**a**) shows a halo of FB defect emission at 846 nm (1.46 eV).

**Figure 3:**
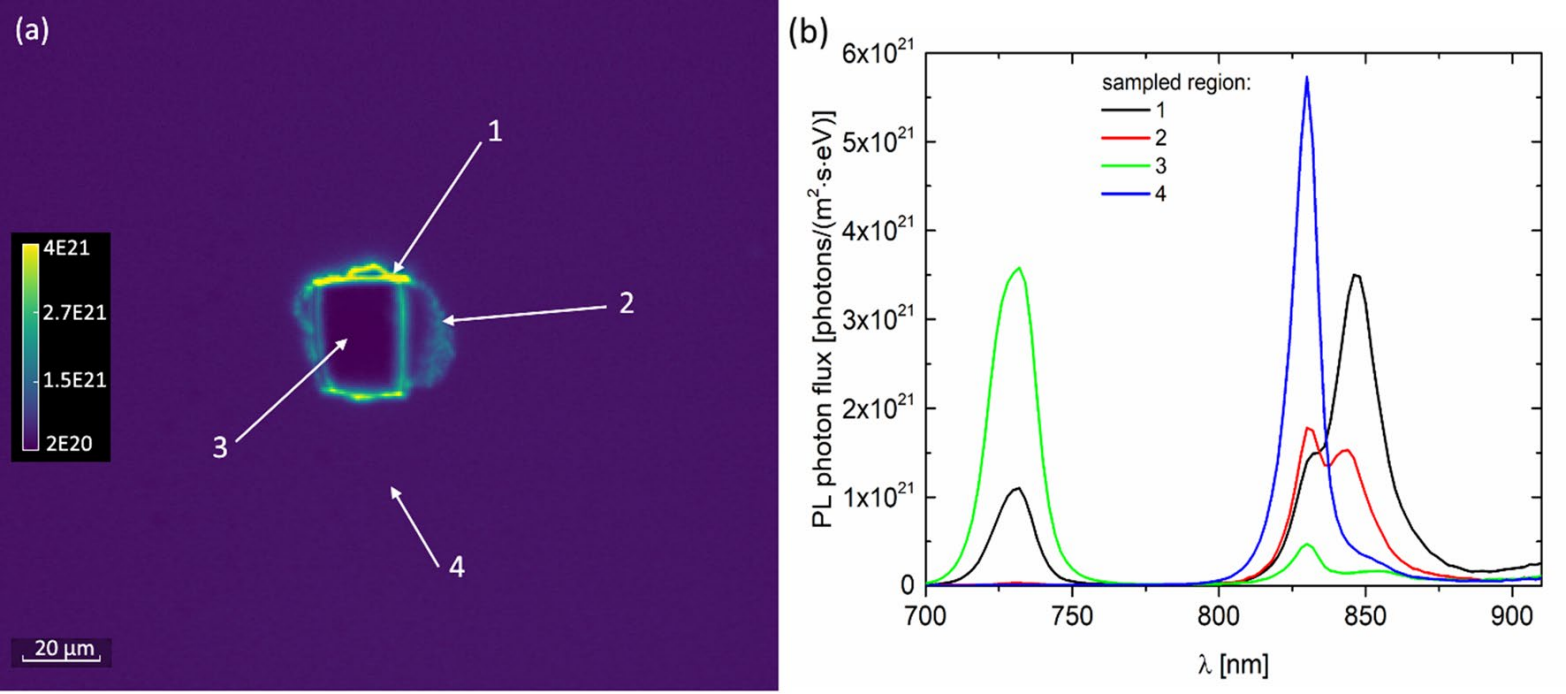
(**a**) A *magnified* single pinhole defect flux map at 846 nm and 77 K. A strong FB defect emission is observed around the edge region of the pinhole. (**b**) A sampling of the full PL spectra at several spots as labeled in (**a**).

Figure 2a,b show side by side PL images of the same region at T = 77 K and wavelengths of 730 nm and 846 nm (1.465 eV) corresponding to the peak wavelengths of the DBR and the FB luminescence, respectively. First, it appears that almost all pinhole defects do result in the formation of the 846 nm FB peak but the reverse is not always the case, as shown by labeled defects *C* and *D*, which only appear at 846 nm. Therefore, pinhole defects, which may be caused during processing of the devices, are not the sole cause of the FB defects. Second, the FB luminescence peak appears as a halo around the edges of the pinhole. This observation is shown more clearly in Fig. 3a, where we have magnified one of these pinhole defects with a 20 × objective. This image, taken at 77 K and 846 nm clearly shows a halo region around the pinhole site where a strong PL signal is observed. Samples of the full emission spectra at several labeled spots are shown in Fig. 3b. The region around the upper edge of the defect, i.e., spot 1, gives the strongest signal but the BB peak is still present. A location like spot 2 shows an equal mix of BB and FB peaks while a spot like 4 is clearly dominated by the BB peak alone. Fig. S2 in the “Supplemental Data” shows a larger region around this defect at 3 different wavelengths. A smaller halo-like defect (≈ 10 µm in diameter) is visible on the lower right corner of that image with characteristics identical to the larger defect (≈ 30 µm). We also show an image from another device showing two nearby, halo-like defects emitting a peak signal at 846 nm with similar characteristics.

The mechanisms by which these pinholes form in the GaAs layer remain unclear. One possible explanation is that a defect nucleation site formed during the epitaxial growth of the DBR layer propagates through all the subsequently grown material^26^. Another possible explanation is that a hole or a bubble was present in the photoresist layer during the mesa etch process, causing all the exposed semiconductor surfaces to be etched down to the DBR layer since that layer is the target mesa etch depth.

### The band-to-band photoluminescence analysis

**Figure 4:**
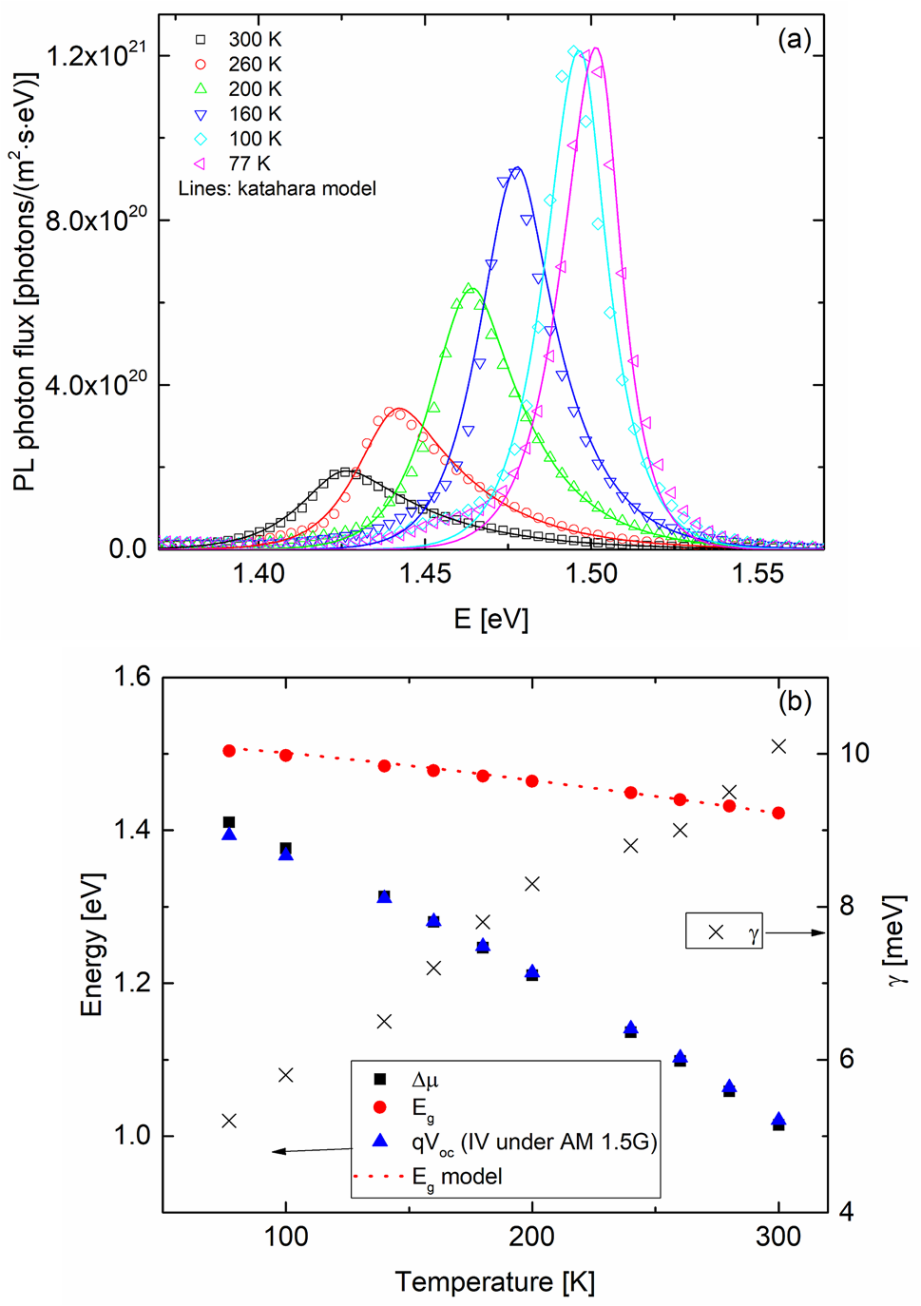
(**a**) A sampling of the absolute photon flux spectra of the BB transition at several temperature*s*, extracted from a large field of view (3.5 mm). *Solid* lines are Katahara model *calculations*. (**b**) The temperature dependence of the fit parameters, plus the qV_oc_ measurements under AM 1.5 G.

The full temperature dependencies of the FB and BB peak intensities and maximum peak energies reveal significant information regarding the physics governing these optical transitions^27^. We first start with the BB peak analysis. We extracted the full BB spectra at several temperatures between 300 and 77 K from across large areas of the devices, i.e., ≈ 3 mm × 3 mm FOV. A sampling of these absolute spectra is shown in Fig. 4a at 2 nm intervals. The total photon flux (photons/m^2^ s) was calculated by integrating the area under each curve. As T is lowered, the total photon flux increases linearly with inverse T until it saturates for T values below 140 K. A better metric to quantify this observation is the external radiative efficiency (ERE, or sometimes referred to as external luminescence yield) of a solar cell, defined as the ratio of photons emitted to photons absorbed by the system. ERE has been used to understand voltage losses from the ideal V_oc_^28–31^. We approximate the ERE values for each temperature by dividing the total PL photon flux by the incident laser photon flux, which was measured by a calibrated spectroradiometer.

**Figure 5:**
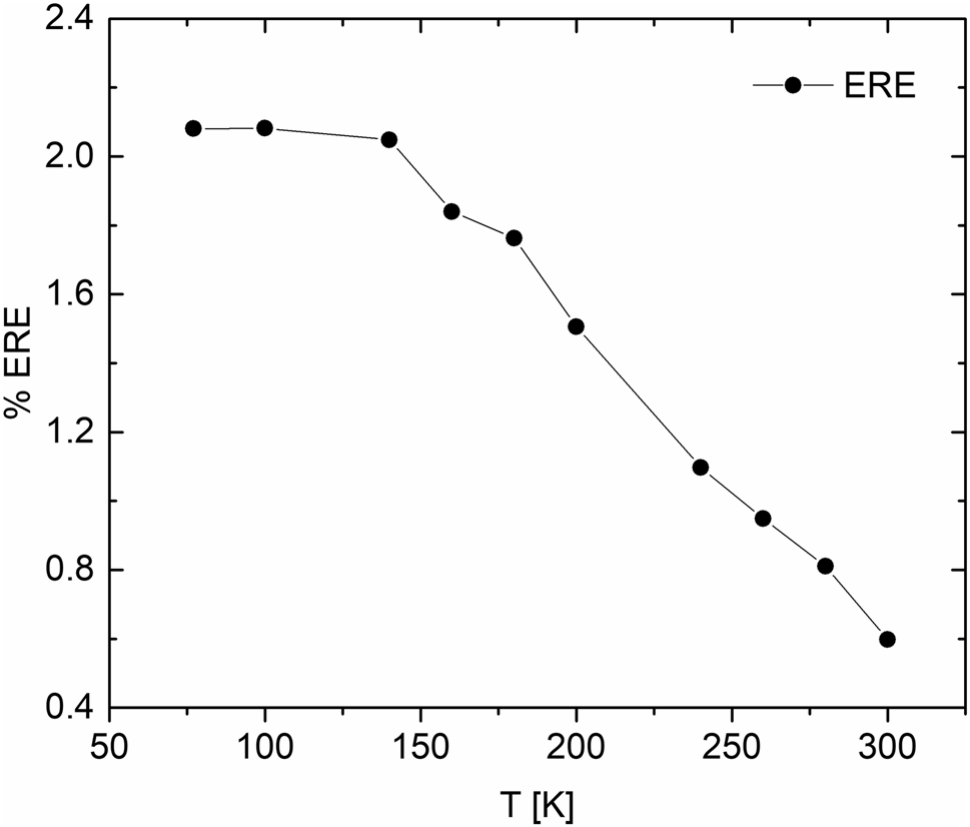
A plot of ERE vs T for the solar cells discussed here. The data are means values across a field of view of ≈ 3.5 mm × 3.5 mm.

A plot of ERE vs. T is shown in Fig. 5, showing a steady increase from 0.6 to ≈ 2% between 300 and 140 K, then saturation at ≈ 2% for temperatures below 140 K. The values reported here for the RJ cells are typical for GaAs cells with a PCE ≈ 22–23%, regardless of shallow or rear junction type. The initial increase in the ERE with 1/T can be attributed to a decrease in the rate of non-radiative recombination in the bulk, particularly the Shockley–Read–Hall (SRH) recombination in GaAs^32^. As T is lowered further, SRH recombination decreases significantly, and radiative recombination becomes the dominant mechanism in the active layer. In the radiative limit, the ERE value can still fall significantly short of 100% (i.e., the Shockley limit), impeded by other non-radiative losses such as absorption into the substrate due to an imperfect back reflector, recombination at the window/emitter interface and other factors^22,31^. Improving the ERE value beyond the observed 2% will require fine-tuning of device architecture to maximize light extraction.

Next, we used a recent model of PL with unified band edge and sub-band gap absorption through a joint DOS function^21^ to fit our PL curves (a visual fit) and extract the fit parameters *Δµ* (quasi Fermi level splitting energy), *γ* (disorder energy of the tail states), and *E*_*g*_. The parameter *γ* captures the broadening of the low energy PL tail, with a larger *γ* corresponding to more absorption and emission in that region, hence a broader PL lineshape. We refer to this model, which is expressed in more detail in “Methods”, as the Katahara model. In general, the Katahara model captures the lineshape of our PL spectra very well, particularly at higher temperatures, as shown by the solid line fits. Fit parameters have high sensitivity and low estimated uncertainty values as described in “Methods”. In the very low T regime, such as the 77 K data shown in Fig. 4a, a small bump appears on the low energy tail region at 1.46 eV which is indeed the peak energy of the FB emission observed from localized defects. Therefore, the FB defects are present throughout the whole active layer, albeit at much lower concentrations such that their PL response remains suppressed at high temperatures and only slightly shows up at lowest T measurements.

The Katahara model fit parameters are plotted in Fig. 4b as a function of T, along with the GaAs accepted E_g_(T) model from the literature^33^ and also the V_oc_ of this device under the air mass 1.5 G illumination condition, as measured under a solar simulator. The laser illumination intensity (532 nm laser, total irradiance ≈ 620 W/m^2^) produces a similar incident photon flux on the device as the solar simulator under 1-sun illumination (≈ 2 × 10^21^ photons/m^2^s); therefore, the separate V_oc_ measurements from a wired cell agree remarkably well with *Δµ* values from PL. This agreement confirms that absolute PL can be used as a method to predict the final device V_oc_ if ohmic, low resistance contacts can be established^34^. The E_g_ parameter also agrees exceptionally well with the E_g_(T) model of Eq. (9) in “Methods”. The *γ* parameter is reduced with decreasing temperature, giving rise to a sharpened PL lineshape. As T is lowered, the reduction of the lattice thermal energy likely causes a freezing of the low energy tail states where trapped carriers are unable to participate in BB recombination, effectively reducing the disorder energy within this model.

### The FB defect photoluminescence peak

**Figure 6:**
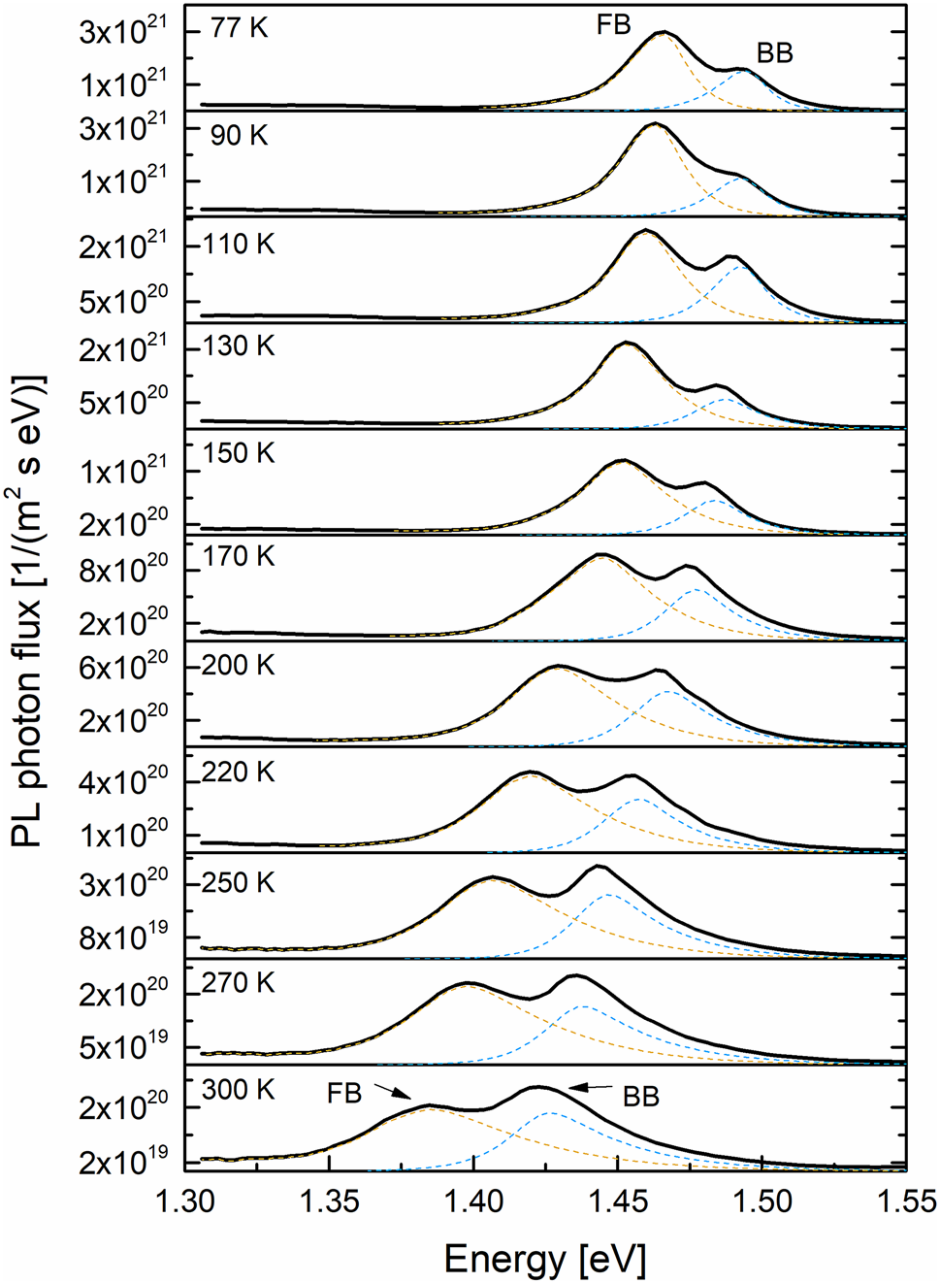
The absolute PL photon flux at several temperatures from the location marked as “spot 1” in Fig. 3. The dashed orange and blue lines are the deconvoluted FB and BB individual peaks.

**Figure 7:**
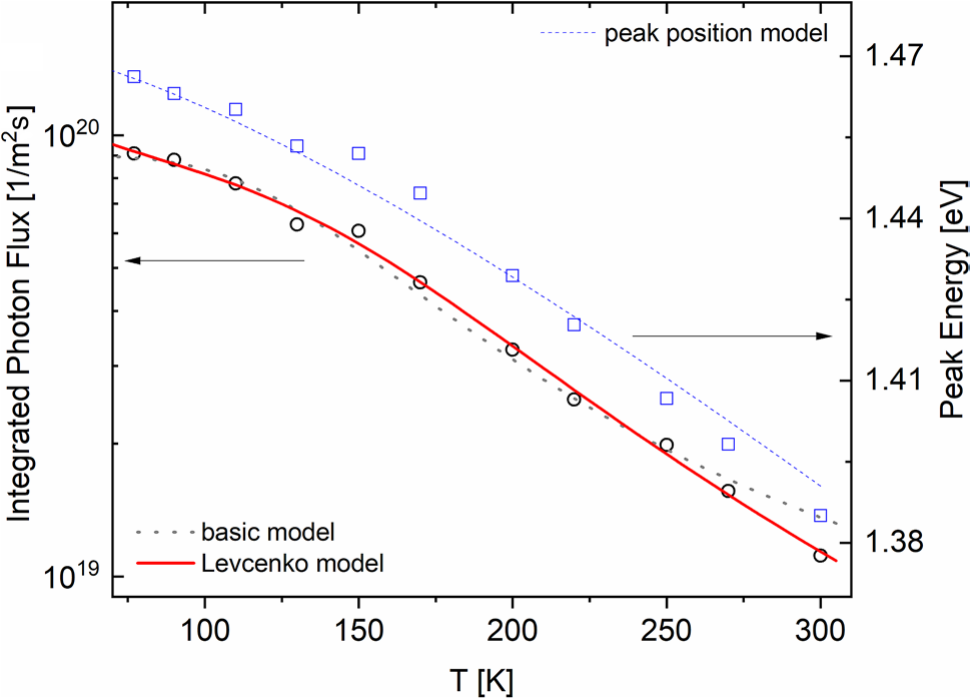
The total FB flux and max peak energy as a function of temperature for the data in Fig. 5. The dotted dark curve is the least square fit of Eq. (3) to the data and the solid red curve is the best fit based on Eq. (4). The blue dotted curve is the E_peak_(T) fit to the peak energy data. The Levcenko model best fit parameters are: *α* = 0.35, *E*_*A*_ = 41.3 meV, *A* = 4.2 × 10^20^ m^-2^ s^-1^ and the *C* = 0.0038.

We next turn to the FB results. As discussed earlier, the FB emission is usually observed around the pinhole sites, but there are other non-pinhole defects, such as spots C and D in Fig. 2b where the FB signal can be observed. Although not certain, these defects may have originated in the high growth layers. Figure 6 shows the temperature evolution of the PL emission near spot 1 in Fig. 3a where the FB signal was significant even at T = 300 K. As T is lowered, both the FB and BB max peak energies shift towards higher energy and the absolute magnitude of both peaks increase as well. However, FB intensity becomes dominant over BB as T is reduced, suggesting that FB recombination is fundamentally different than band-to-band recombination. We separated these double peaks into their constituent single FB and BB peaks, as shown in the dashed orange and blue curves. We used the Katahara lineshape for the BB peak and the following approximation for the FB transition without any consideration for the absorption coefficient and its energy dependence^35^:

1 $$I_{PL}^{FB} = C\sqrt {E - E_{g} (T) + E_{A} } \exp \left(\frac{{ - (E - E_{g} (T) + E_{A} )}}{kT} \right)$$

here, *C* is a constant, *E*_*g*_(*T*) is given by Eq. (9), *k* is the Boltzmann constant, and *E*_*A*_ is the activation energy of an impurity state. Equation (1) only works well in describing the FB lineshape for the max peak and the higher energy tail. The low energy sub-band gap tail would require a treatment similar to Katahara’s treatment of the band-to-band emission and we simply used the actual tail data to fully reconstruct the FB emission curves. With the two separate PL lineshapes fully resolved, we integrated the area under the FB curve to obtain the total photon flux and plotted it as a function of T in Fig. 7. We also plot the max peak energy as a function of temperature in Fig. 7 (right y axis).

The peak position of the FB transition is obtained by differentiating Eq. (1), setting it equal to 0 and solving for *E*, giving:

2 $$E_{{{\text{peak}}}} (T) = E_{g} (T) - E_{A} + kT/2.$$

For the temperature dependence of the FB intensity (or flux), several treatments have been previously considered^27,36,37^. The most basic treatment assumes thermal emission of trapped carriers between localized shallow states within the gap and the conduction or valance band, with the PL flux having a dependence given by^27^:

3 $$I_{PL,tot}^{FB} = \frac{A}{{1 + C\exp ( - E_{A} /kT)}},$$

where *A* and *C* are constants. In Fig. 7, we fit this relationship to our FB data, which gives a best fit activation energy of 56.5 ± 3.7 meV. On the other hand, the *E*_peak_ relationship above (Eq. 2) predicts a best fit activation energy of 45 meV ± 1.1 as shown by the dashed blue curve.

We find that a better agreement with our data is achieved through a more complete treatment by Levcenko^36^. In this model, the impurity atoms form a single energy band within the gap where several processes can take place. Assuming an acceptor level near the valance band, (a) a free hole can be captured by the impurity level. (b) a trapped hole at the impurity level can be recombined with a free conduction band electron. (c) trapped holes can be thermally ionized to the valence band. The total integrated photon flux of such a free-to-bound transition gives the following temperature dependence:

4 $$I_{PL,tot}^{FB,Levc} (T) = \frac{{AT^{ - \alpha } }}{{1 + CT^{3/2} \exp ( - E_{A} /kT)}}$$

where *A* is a constant, *C* is related to the ratio of the effective DOS to carrier density and assumed to be temperature independent over a limited range, and *α* is a fixed exponent. A fit of Eq. (4) to our data is shown by the red solid curve in Fig. 6 and shows an excellent fit. The *E*_*A*_ value from the fit of this model to our data is 41.3 ± 6.1 meV, which is in close agreement with the value extracted from Eq. (2).

We considered the possibility that the observed 1.46 eV defect emission could be related to a donor–acceptor pair recombination. In addition to a different behavior of PL intensity with T^27^, such transitions have also been shown to exhibit a substantial dependence of the peak PL position with incident laser intensity with the peak position shifting to higher energies (a blue shift) as the intensity is increased^38^. As shown in Fig. S4 in the “Supplemental information”, we do not observe a noticeable change in the peak position with incident laser intensity over two orders of magnitude of intensity change. Therefore, we conclude that a free-to-bound optical transition is the most likely explanation for the secondary PL emission observed at 1.46 eV at 77 K.

Although the intensity of the FB PL signal is likely an indication of the relative magnitude of the localized defect density, it is hard to quantify it with the characterization tools currently available. In terms of the effect of such localized defects on carrier lifetimes, we do not expect a global effect since each cell typically only shows a handful of such defects. However, local carrier lifetimes in the vicinity of these defects are likely affected because the BB photon flux at defect sites is generally lower than other parts of the cell. This observation points to increased non-radiative recombination at the defect sites in addition to the FB radiative emission. Microscale time-resolved PL imaging could potentially reveal subtle differences in carrier lifetimes in the vicinity of such defects^39,40^.

### Defect identification

Several studies have reported the presence of a defect transition in the energy range 1.45–1.47 eV in n-type, Si-doped GaAs substrates and films at 77 K and have associated this transition to either Ga antisite defects, Ga_As_, or Ga vacancies, V_Ga_, acting as deep acceptors^14–16^. In such a scenario, free electrons from the conduction band can recombine radiatively with holes trapped on these acceptor sites, leading to the radiative transitions observed. The exact peak position depends on the dopant concentration with higher concentrations resulting in a higher energy shift. The localized radiative defect peak at 1.46 eV in our devices is consistent with these previous reports and appear at this energy throughout the active region. A recent temperature dependent study in a highly doped GaAs substrate confirms our temperature measurement trends^16^ and the identification of this peak as a free-to-bound transition. A question that remains is why the area around the pinhole defects become sites of Ga antisite defects in such high concentrations that result in a strong luminescence signal. Disruptions in the lattice parameters near the pinholes could result in formation of arsenic vacancies that are energetically favored to form both Ga vacancies and Ga_As_ antisite species in n-type GaAs^14^. Ultimately, structural characterization techniques such as transmission electron microscopy and scanning electron microscopy with focused ion beam are probably best suited to answer this question since they can provide atomic-scale resolution.

In conclusion, we have used wide-field, high resolution absolute hyperspectral photoluminescence imaging to study the band-to-band emission and localized free-to-bound radiative defects in the active layer of rear-junction GaAs solar cells. High magnification imaging reveals local halo-like regions of strong sub-band gap emission around pinhole sites within the GaAs emitter layer. Although the physics of free-to-bound transitions in GaAs is well-known, local detection of such phenomena are only possible with a high-resolution imaging technique such as with the hyperspectral imaging instrument we have utilized for this study. The temperature dependence of the photoluminescence flux from these defect regions shows a behavior consistent with optical transitions between free electrons and trapped holes on impurity acceptor sites with an activation energy of about 41 meV. Formation of high concentrations of gallium antisite or vacancy defects at the site of the pinholes is the most likely explanation regarding the chemical nature of these defects. The techniques presented in this paper may be invaluable to achieving defect-free III–V growth at extremely high growth rates by providing unique spatial information about defects with very high resolution. The details provided from such absolute measurements have the potential to inform improved growth strategies and higher device performance.

## Methods

### Device fabrication and structure

The devices were grown using a K475i Veeco MOCVD tool (Certain commercial equipment, instruments, software, or materials are identified in this paper to specify the experimental procedure adequately. Such identification is not intended to imply recommendation or endorsement by the National Institute of Standards and Technology, nor is it intended to imply that the materials or equipment identified are necessarily the best available for the purpose.). The precursors were trimethylgallium (TMGa), trimethylindium (TMIn), trimethylaluminum (TMAl), arsine (AsH_3_), phosphine (PH_3_), disilane (Si_2_H_6_), diethyltellurium (DETe), carbon tetrabromide (CBr_4_), and dimethylzinc (DMZn). GaAs emitter and base growth rate was approximately 1 μm/min. The GaAs V/III ratio was 9. Growth temperature varied between 640 to 680 °C, and chamber pressure was held at 42 Torr. GaAs substrates were (100) 5° offcut toward <011> p-type. Solar cells were grown above an AlGaAs distributed Bragg reflector (DBR) with a stop-band nominally centered at 850 nm. Devices were fabricated with standard lithographic and wet etch processes into mesas of 5 × 5 mm^2^. Device structure is shown in Fig. 1a. Further structure details and process information can be found in reference^18^.

### Device characterization

The solar cells were wire-bonded and characterized using current vs. voltage and EQE measurements prior to HS imaging. The typical V_oc_ and the short circuit current I_sc_ under air mass 1.5 G illumination for these cells were 1.032 V and 26.8 mA/cm^2^, respectively, with the power conversion efficiency (PCE) at ≈ 22.55% (See Table S1 in the “Supplemental”) but higher performance has been reported previously^18,20^. The EQE at 840 nm is 93.7% (See Fig. S3 in the “Supplemental Information” for the whole EQE curve). The V_oc_ as a function of temperature was also measured on a wired sample inside an optical cryostat (see below) under the solar simulator from 300 to 77 K.

### Hyperspectral imaging

HS imaging in PL mode was performed using a wide-field imaging system by Photon ETC (Certain commercial equipment, instruments, software, or materials are identified in this paper to specify the experimental procedure adequately. Such identification is not intended to imply recommendation or endorsement by the National Institute of Standards and Technology, nor is it intended to imply that the materials or equipment identified are necessarily the best available for the purpose.), with capability to scan the spectral region of 400–1600 nm using two camera systems. Although HS imaging in EL mode was also possible for these cells, we opted for PL instead because we wanted to analyze our luminescence emission data within the framework of the PL model developed by Katahara. Furthermore, since no wiring of cells are required with PL, more device areas can be examined during one cryostat cool down cycle. Several microscope objectives allow for a FOV ranging from ≈ 4 mm to less than 500 µm. The highest spatial resolution of ≈ 1 µm was achieved with a magnification of 20×. In the 400–1000 nm region, the spectral resolution is better than 2 nm. For PL excitation, a 532 nm laser is used to uniformly illuminate the cell, with intensities ranging from 70 mW/cm^2^ to ~ 4 W/cm^2^, depending on the objective and laser setting. The reported data here are all at the lowest intensity settings to reduce heating effects. The absolute calibration of the imager to obtain absolute photon flux rates is discussed elsewhere^10,41^. Temperature dependent imaging was performed with a liquid nitrogen-flow optical cryostat under vacuum from 300 to 77 K. Several cells were investigated for this work and the phenomena discussed here were universal in all samples. The relative uncertainty of the PL flux rates presented here are about ± 15%. Wavelength calibration of the HS system was performed with pen lights and is accurate to within 1 nm.

### The Katahara model

The joint DOS as a function of energy, *E*, is given by^21^:

5 $$\begin{aligned} G(E) = \frac{1}{{\gamma 2\Gamma (1 + 1/\theta )}}\times \int\limits_{{ - \infty }}^{{E - E_{g} }} {\left( {\exp \left( { - \left| {\frac{u}{\gamma }} \right|^{\theta } } \right)\sqrt {(E - E_{g} ) - u} } \right)} {\text{ d}}u \\ \end{aligned}$$

where *γ* is the disordered energy parameter, *θ* is an exponent fixed at 1.1, and Γ is the gamma function. From this expression, the absorption coefficient, *α(E)* within the GaAs active layer is given by:

6 $$\alpha (E) = \alpha_{0} G(E)\left( {1 - \frac{2}{\exp ((E - \Delta \mu )/2kT) + 1}} \right)$$

where *T* is temperature, α_0_ is the absorption coefficient near the band edge here set to 4.1 × 10^4^/cm for all T, and *k* is the Boltzmann constant. For an absorptivity model within our unique device geometry, we use an incoherent cavity approximation where we treat the device structure like a Fabry Perot cavity with reflectivity coefficient *R*_*1*_ for the top antireflection coating and *R*_*2*_ for the bottom DBR layer. In this case, absorptivity *a(E)* is given by^33^:

7 $$\begin{aligned} a(E) & = 1 - R_{1} \\ & \quad - \frac{{(1 - R_{1} )^{2} R_{2} \exp ( - 2\alpha d) + (1 - R_{1} )(1 - R_{2} )\exp ( - \alpha d)}}{{1 - R_{1} R_{2} \exp ( - 2\alpha d)}} \\ \end{aligned}$$

where *d* is the diffusion length and *R*_*1*_ and *R*_*2*_ were calculated as a function of *E* based on the optical parameters of our device layers. The PL photon flux, *I*_PL_, is given by:

8 $$I_{PL} (E) = \frac{2\pi }{{c^{2} h^{3} }}\frac{{E^{2} a(E)}}{\exp ((E - \Delta \mu )/kT) - 1}$$

where *c* is the speed of light and *h* is the Planck’s constant. Equations (5) and (8) are solved numerically and visually fit to the individual PL spectra at different temperatures with the fit parameters being Δ*µ*, *γ* and *E*_*g*_. Our estimated uncertainty for these fit parameters are 1.5 meV, 0.5 meV and 2 meV, respectively. With regard to the band gap energy E_g_, an initial estimate was first calculated based on the relationship^33^:

9 $$E_{g}^{GaAs} (T) = 1.519 - \frac{{5.4 \times 10^{ - 4} T^{2} }}{T + 204}$$

Then, this estimate was allowed to slightly change in order to produce a good fit to the PL curve. Parameter *d* was fixed at 5.7 × 10^–4^ cm for all *T* because it has negligible temperature dependence over the range probed here.

## Supplementary Information

Supplementary Information.

## Data Availability

The datasets used and/or analyzed during the current study are available from the corresponding author on reasonable request.
