# Supplementary material for: Visualizing localized, radiative defects in GaAs solar cells
Source: Sci Rep. 2022 Sep 1;12:14838. doi: 10.1038/s41598-022-19187-4 (PMC9436936; doi:10.1038/s41598-022-19187-4)
Supplement: Supplementary file 1 — Supplementary Information. [file 41598_2022_19187_MOESM1_ESM.pdf]

Supplementary Information

Visualizing Localized, Radiative Defects in GaAs Solar Cells

Behrang H. Hamadani[1*], Margaret A. Stevens[2], Brianna Conrad[1], Matthew P. Lumb[3], and Kenneth J. Schmieder[4]

[1]National Institute of Standards and Technology, Gaithersburg, MD 20899 USA
[2]NRC Postdoc Residing at NRL, Washington, DC 20375 USA
[3]Formerly with George Washington University, Washington, DC 20052 USA
[4]U.S. Naval Research Laboratory, Washington, DC 20375 USA

I. The photoluminescence signal of DBR-only films

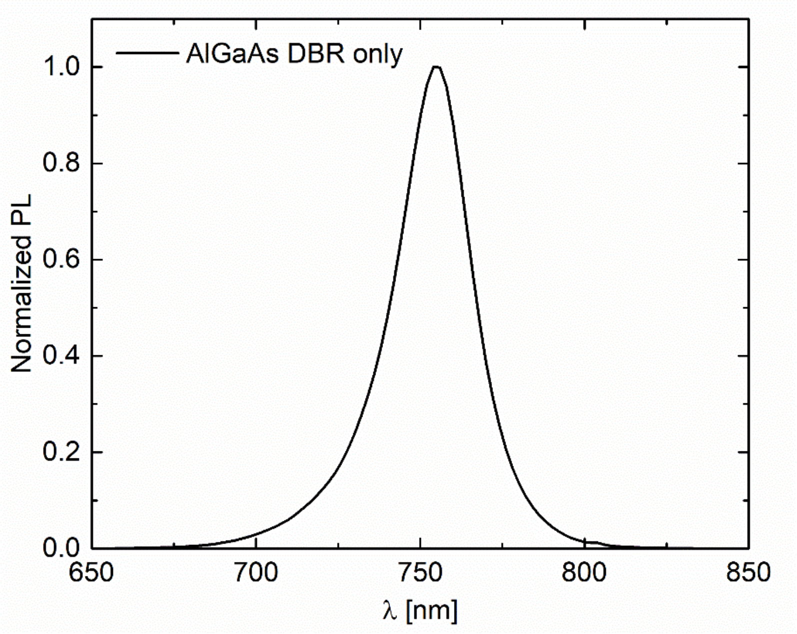

Fig. S1. The normalized PL signal observed from an AlGaAs DBR layer grown on a GaAs substrate. The peak energy and lineshape matches closely with the PL signal observed through the pinhole defects, indicating that the pinhole defects reach all the way through to the DBR layer.

II. PL images at 77 K

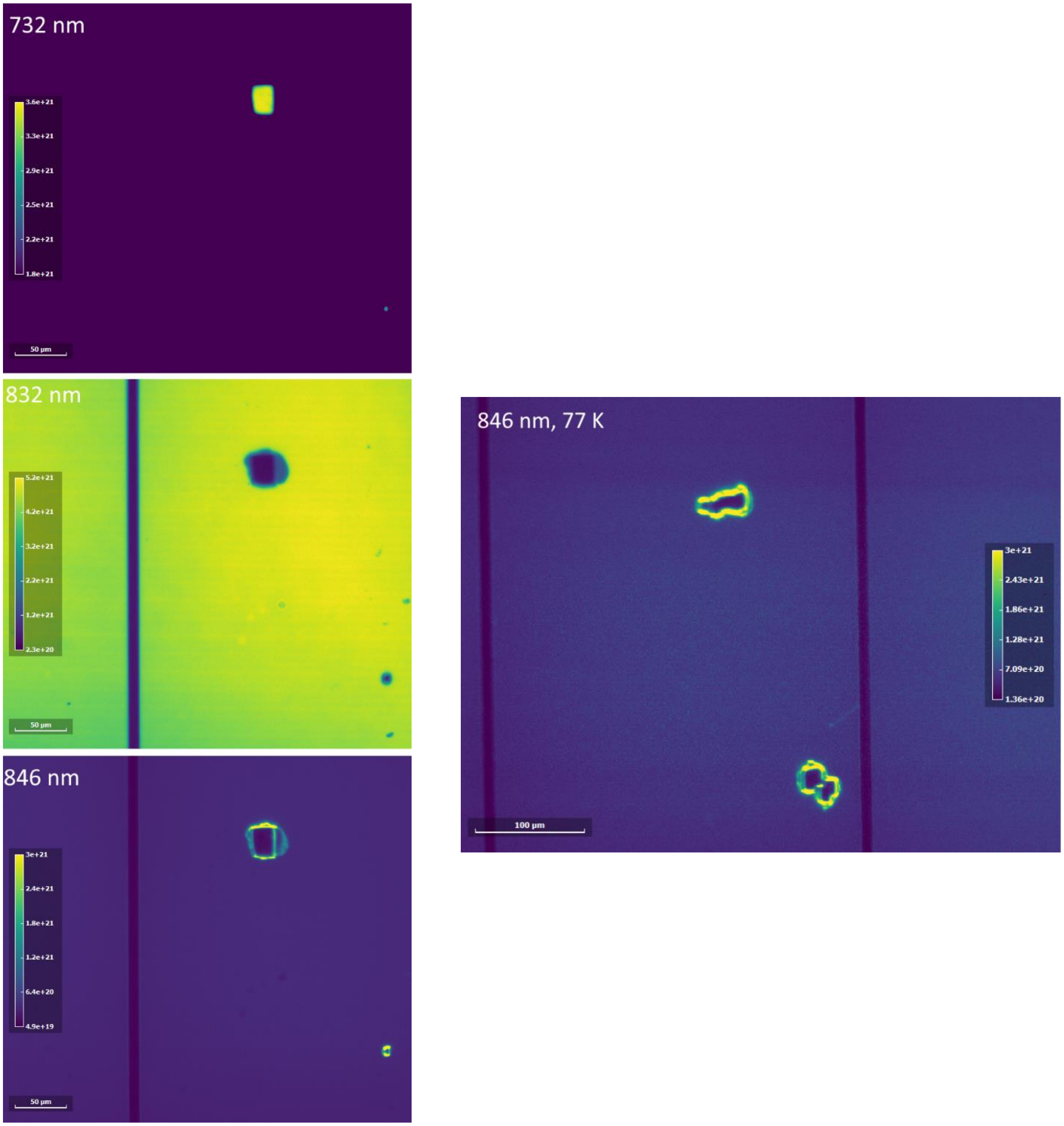

Fig. S2. (left) The absolute PL emission maps (photons/m^2 s eV) of a region of the cell with two local defects showing the 846 nm FB signal as a halo around two processing defects, one large and one small, at 77 K. (right) PL signal at 846 nm from a section of another device showing two nearby defect sites with a halo-like FB emission.

III. Electrical performance parameters and the EQE of the RJ GaAs solar cells

Table S1- The measured J-V curve parameters of a typical GaAs solar cell employed for this study

Input Condition (T~25° C)	V_{oc} (V)	Jsc(mA/cm^2)	Pmax (mW)	Vmax (V)	Jmax (mA/cm^2)	Fill Factor	% PCE
AM 1.5G-1000 W/m^2	1.032	26.805	5.636	0.885	25.481	0.815	22.545

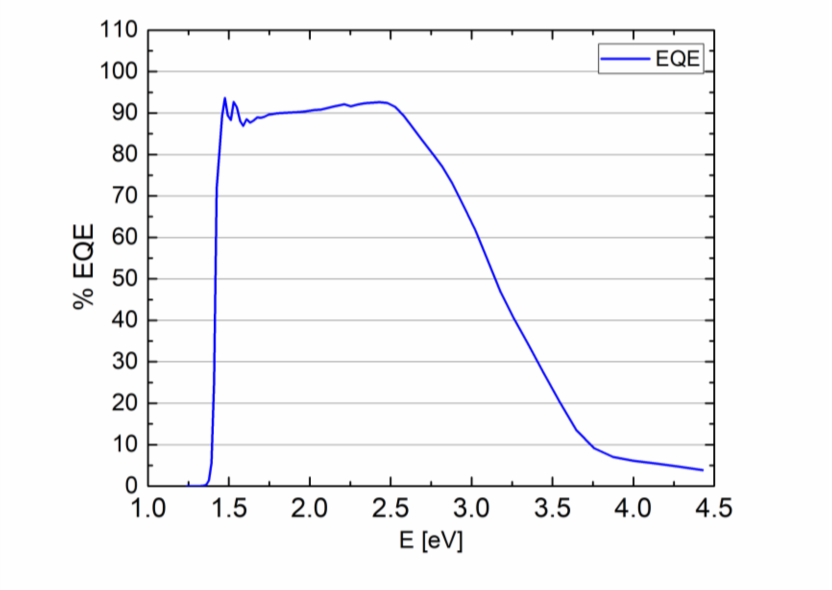

Fig. S3. The absolute EQE of a typical RJ GaAs solar cell used for this study.

IV. Laser intensity dependence of the FB transition

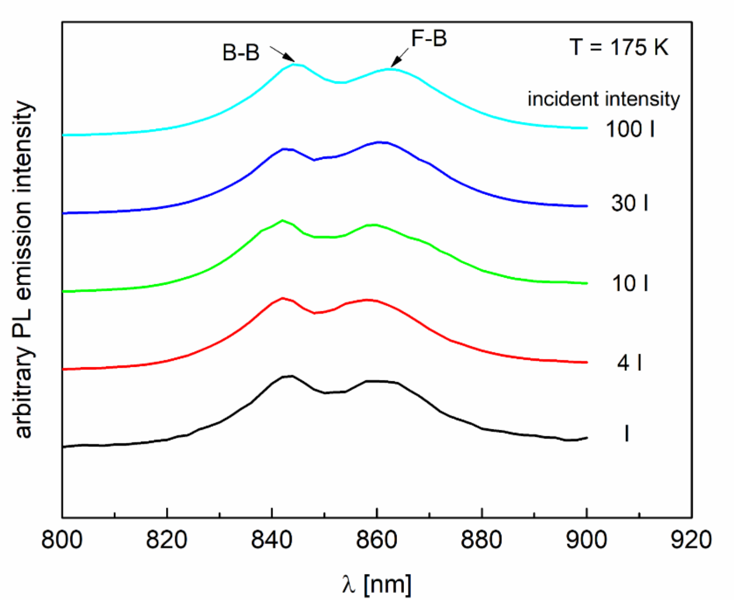

Fig. S4. Normalized PL spectra at 175 K from a localized defect region at several incident laser excitation intensities The peak positions of the identified FB or BB peaks show no noticeable change with intensity, therefore eliminating the possibility that the FB defect is a donor-acceptor transition.
